# Vascular Access 4.0 for Hemodialysis: Toward a Needle-Free, Smart, Closed, and Connected System

**DOI:** 10.3390/jcm15031144

**Published:** 2026-02-02

**Authors:** Bernard Canaud, Hafedh Fessi, Michael Rys, Eric Jean, Ludovic Canaud

**Affiliations:** 1 School of Medicine, Montpellier University, 34090 Montpellier, France; 2 Nephrology & Dialysis Department, Hôpital Le Mans—University Paris Sorbonne, 75006 Paris, France; hafedh.fessi@bbox.fr; 3 SÆPTUM SAS, 14200 Hérouville-Saint-Clair, France; michael.rys@saeptum.com (M.R.); eric.jean@saeptum.com (E.J.); 4 Vascular and Thoracic Surgery Department, University Hospital Arnaud de Villeneuve, CHU Montpellier, 34295 Montpellier, France; l-canaud@chu-montpellier.fr

**Keywords:** end-stage kidney disease, hemodialysis, kidney replacement therapy, vascular access

## Abstract

Vascular access remains the cornerstone of effective hemodialysis but also constitutes a major source of burden, including dysfunctions, infections, patient discomfort, and other access-related morbidities. As dialysis care evolves, there is a pressing need to move beyond conventional approaches, marked by repeated needle punctures and open connection systems, toward safer, more comfortable, and technologically advanced solutions. This narrative article presents a forward-looking vision of vascular access connectivity supported in current clinical and technological knowledge. It explores how emerging connectivity, particularly needle-free port systems, could reshape the future of dialysis care. We briefly review existing vascular access modalities, including central venous catheters (CVCs) and arteriovenous (AV) accesses, along with their associated limitations. Special focus is given to the burden of infection, patient-reported discomfort, and workflow inefficiencies. We then examine emerging closed-system technologies designed to reduce contamination risk, improve patient experience, and potentially support long-term clinical outcomes. Drawing on advances in material science, biomedical engineering, and infection prevention, we outline a forward-looking vision for vascular access that aligns with patient-centered care, facilitates home-based treatment and remote connectivity, and anticipates future developments, such as wearable artificial kidneys within a value-based healthcare framework. However, the clinical adoption of these new technologies will require careful evaluation of long-term safety, durability, cost-effectiveness, training requirements, and real-world performance, underscoring the need to balance innovation-driven benefits against practical, regulatory, and organizational challenges.

## 1. Introduction

Vascular access (VA) is the cornerstone of extracorporeal therapies and remains the Achilles’ heel of chronic kidney disease patients undergoing kidney replacement therapy by hemodialysis and related modalities [1,2]. Despite continuous innovation, current VA options remain largely limited to arteriovenous (AV) fistulas, either using native vessels or synthetic grafts, and central venous catheters for venovenous access [1,3,4]. The most recent advancement has been the development of bioengineered vascular grafts, which have shown promising preliminary results but remain in the experimental stage [5,6,7,8,9]. Within this context, and despite limitations related to maturation and initial usability, the native AV fistula continues to represent the reference, owing to its superior performance, longer patency, improved patient outcomes, and greater cost-effectiveness compared with other access types [10,11].

Nonetheless, AV fistula use is not without challenges [12]. Cannulation can be associated with pain, anxiety, and technical difficulty and may lead to complications such as needle dislodgement, hematoma, bleeding, or infection [10,11,13]. To address these issues, several port-based connection systems have been developed with the aim of simplifying and securing vascular access while improving patient comfort and satisfaction [14,15,16,17,18]. However, most have failed to achieve sustained clinical adoption because of technical limitations, complication rates, particularly infection, or cost constraints [16,17,19,20].

In this narrative article, we briefly review existing vascular access modalities and their intrinsic limitations, analyze earlier port-based devices and lessons learned from past failures, and explore how emerging connectivity-enabled solutions, particularly needleless, smart, closed, and remotely connected (Vascular Access 4.0), could transform the future of dialysis care. This topic is especially timely given the convergence of miniaturized sensors, digital health platforms, wearable technologies, and the expansion of home and self-care hemodialysis, which together are redefining how vascular access can be monitored, protected, and integrated into the care ecosystem. Unlike previous reviews that focused primarily on materials, patency, or infection risk, this review adopts a systems-level and forward-looking perspective, positioning vascular access as a connected therapeutic interface rather than a passive device. We discuss the clinical, technological, and organizational implications for future clinical adoption and healthcare system evolution.

## 2. Current Vascular Access Modalities and Their Limitations

Current vascular access relies primarily on two main approaches: venovenous access using central venous catheters (CVCs) and arteriovenous access using fistulas or grafts [21,22,23,24].

**Venovenous access** involves the insertion of a CVC, most commonly into the internal jugular vein for tunneled catheters or the femoral vein for temporary non-tunneled CVCs. Alternative routes, such as subclavian vein or translumbar vena cava access, are used only as rescue options when standard sites are no longer viable [22,23,24]. Despite their ease of use and immediate functionality, CVCs are associated with significant drawbacks, including frequent dysfunction and thrombosis, a high risk of infection, and central venous stenosis, which may reduce dialysis efficiency.

**Arteriovenous access** remains the preferred long-term solution and can be achieved by creating a native-vessel (autologous) arteriovenous fistula (AVF) using the patient’s own vessels or by interposing a synthetic graft (arteriovenous graft, AVG) between an artery and a vein [22,23,24]. The most frequent anatomical locations are on the forearm and upper arm, while femoral sites are rarely indicated due to higher complication risks. Grafts are most commonly made from expanded polytetrafluoroethylene (ePTFE) and only rarely from heterologous or biologic grafts (e.g., cryopreserved saphenous veins).

**Percutaneous or endovascular procedures** to create forearm arteriovenous fistulas (e.g., Ellipsys and WavelinQ) have emerged as minimally invasive alternatives to surgery and report encouraging technical success, maturation, and 1–2-year patency in observational cohorts and multicenter series [25,26]. Nevertheless, head-to-head comparative evidence versus standard surgical AVF remains limited, and major guidance does not currently endorse endovascular AVF as a routine first-line option [22,23].

In recent years, **bioengineered vascular prostheses** have emerged as a novel option, designed to combine the durability of synthetic materials with the biocompatibility of native vessels [7,8]. Early pilot and clinical studies have shown encouraging results in terms of patency and reduced complication rates, including infection risk. However, these devices remain under investigation and are not widely available for routine clinical use.

### 2.1. Venovenous Access Systems

**Venovenous access** relies on the insertion of central venous catheters (CVCs), which are primarily indicated in patients with end-stage kidney disease (ESKD) who require urgent start initiation of dialysis in the absence of a mature arteriovenous access, or as a temporary rescue option following permanent access failure [21,22,23,24].

**Temporary (non-tunneled) double lumen CVCs** are typically inserted into the femoral vein and intended for very short-term use to initiate hemodialysis in unplanned or “crash-landing” ESKD patients. In contrast, tunneled double-lumen CVCs, most commonly placed in the internal jugular vein, are used as a bridging solution while awaiting creation or maturation of a permanent arteriovenous access [27]. Temporary CVCs offer limited dialysis performance and are suitable only for brief use, being associated with a high rate of complications [28].

**Tunneled CVCs** provide improved functionality, including higher achievable blood flow rates and dialysis adequacy, depending on catheter design and tip position. However, they remain prone to recurrent dysfunction related to flow limitation, abnormal venous pressures, catheter malposition, thrombosis, or external fibrin sheath formation [29,30,31,32,33,34,35]. These issues frequently necessitate interventional procedures or the use of fibrinolytic locking solutions to maintain patency [36].

**The risk of catheter-related bloodstream infection (CRBSI)** is reported to be three- to seven-fold higher than that observed with arteriovenous access [37,38,39]. Moreover, CVC use predisposes patients to central vein stenosis or thrombosis, which may compromise future vascular access options.

**From an operational standpoint**, CVC handling is more resource-intensive than arteriovenous access [40]. CVC connection and disconnection require strict aseptic conditions, including sterile gowns, gloves, cap, and drapes, and often involve two operators, one managing the dialysis machine and another handling the catheter [39]. As a result, CVC handling requires approximately 15–20 min per connection/disconnection, compared with 5–10 min for arteriovenous access, and necessitates additional disposable materials, thereby increasing the workload and care-related costs. Although locking solutions have been developed to mitigate thrombosis and infection risk, CVC-based dialysis remains associated with higher overall resource utilization and maintenance costs than AV access [41,42,43,44]. A comparative table (Table 1) summarizing patient experience, workload, and suitability for home or self-care features is provided.

**From the patient’s perspective,** living with a tunneled CVC represents a significant and psychological burden [45]. Daily life is affected by the need for continuous dressing protection, restrictions on bathing or physical activity, and persistent fear of infection. However, paradoxically, many patients find CVCs more comfortable than arteriovenous access because they avoid the pain and anxiety of repeated needling and allow greater freedom of arm movement during dialysis. This may contribute to an improved patient experience and reduced perceived treatment burden. Consequently, once patients experience CVC-based dialysis, they often show reluctance to transition to an arteriovenous fistula despite the well-documented risks associated with long-term catheter use [46].

### 2.2. Arteriovenous Access Systems (Fistulas and Grafts)

**Native Arteriovenous Fistulas:** Arteriovenous access remains the cornerstone of long-term hemodialysis access, offering superior blood flow, dialysis efficiency, lower complications rates, and long-term patency compared to central venous catheters. Two principal types are used in current clinical practice: the native (autologous) arteriovenous fistula (AVF) and the prosthetic arteriovenous graft (AVG). Native Arteriovenous Fistula (AVF): The native AVF is created surgically by directly connecting an artery to a vein, typically in the forearm or upper arm, to provide a high-flow, low-resistance circuit suitable for repeated hemodialysis. The distal radial-cephalic fistula (Brescia–Cimino type) remains the preferred first choice, followed by proximal brachiocephalic or brachiobasilic transposed fistulas when distal sites are exhausted [21,22,24]. The AVF is widely recognized as the gold standard of vascular access because of its long-term patency, lower infection risk, better dialysis efficiency, and cost-effectiveness [10,13]. However, it is also associated with substantial challenges. Maturation failure, defined as the inability of the access to develop adequate flow and diameter for effective cannulation, occurs in up to 30–50% of cases, particularly in elderly, diabetic, and female patients with vascular disease [11]. Contributing factors include small vessel caliber, poor surgical technique, and pre-existing arterial or venous pathology. Even after successful maturation, AVFs are exposed to functional complications, such as stenosis, thrombosis, aneurysm formation, and infection. Regular surveillance and monitoring are required to detect dysfunction early and prevent thrombosis or access loss. In addition, inflammatory and pro-thrombotic biomarkers may help identify patients at increased risk of access failure; among these, the platelet-to-lymphocyte ratio (PLR) has been associated with AVF stenosis and thrombosis [47]. Despite their superior long-term outcomes, these procedures necessitate skilled maintenance and a robust multidisciplinary vascular access program. Primary median survival of native AVF is estimated to be 3 to 4 years after maturation [11,48]. Median access flow measured using the ultrasound (US) dilution method (Transonic^®^) typically ranges from approximately 400 to 600 mL/min in distal access locations and 800 to 1200 mL/min in proximal locations [49,50,51]. Periodic monitoring of access flow and recirculation is recommended by clinical guidelines to detect early access dysfunction, anticipate failure, and enable timely intervention [22,24].

**Prosthetic Arteriovenous Grafts (AVGs):** Prosthetic grafts are used as a secondary option when suitable native vessels are unavailable or when rapid access creation is needed. They typically consist of expanded polytetrafluoroethylene (ePTFE) or other synthetic material interposed between an artery and a vein, most often in the upper arm as loop between the humeral and cephalic or cephalic vein. AVGs offer the advantage of shorter time to cannulation (2–3 weeks versus 6–8 weeks for AVFs) and provide more predictable flow dynamics; however, they are restricted to rope-ladder cannulation and are associated with a higher risk of venous stenosis, subsequent thrombosis, and infection, leading to reduced long-term patency and increased intervention rates [22]. Median survival as primary patency is estimated to be 2 years and then patients require procedural interventions. Median access flow measured in AVG using the ultrasound (US) dilution method (Transonic^®^) typically ranges from 600 to 800 mL/min depending on diameter of graft diameter [52,53,54]. Periodic monitoring of access flow performance is recommended to detect early dysfunction, similarly to AV fistula [22,24].

**Cannulation-Related Burden and Patient Experience:** Cannulation is an essential but highly sensitive aspect of arteriovenous access management [55]. It requires trained staff and standardized needling techniques (rope-ladder, buttonhole, or area puncture), as variability in skill and technique can directly affect access longevity and patient comfort. Repeated needling is associated with pain, anxiety, bruising, hematoma, and pseudoaneurysm formation, representing a major source of patient dissatisfaction and distress. Furthermore, difficult cannulations may increase the risk of access infiltration, bleeding, or early thrombosis. From the patient’s perspective, the AVF, while functionally superior, often represents a daily psychological and physical burden [46]. The repeated trauma and pain of needling, coupled with visible vascular deformities, may negatively affect body image, anxiety, and quality of life. Despite these drawbacks, the AVF remains the preferred access modality because of its proven clinical superiority, durability, and cost-effectiveness compared with grafts or catheters.

**Emerging Developments:** Recent innovations aim to address these limitations through endovascularly created fistulas [56], bioengineered vascular grafts [7], and novel cannulation-assist devices [57,58]. Early clinical series of endovascular AV fistulas report technical success rates of approximately 80–95%, with functional patency at 6–12 months ranging from 60% to 75%, often with reduced surgical trauma and faster recovery compared with surgically created fistulas. Bioengineered vascular grafts have demonstrated promising primary patency rates of ~55–65% at 12 months and low immunogenicity in early trials. Cannulation-assist devices have been associated with reductions in infiltration events and needling-related complications in selected cohorts. However, available data remain limited to small or short-term studies, and further comparative evaluations are required to establish long-term patency, safety, patient-centered outcomes, and cost-effectiveness before widespread adoption [22].

### 2.3. The Overall Vascular Access Burden

Vascular access (VA) remains both the lifeline and the Achilles’ heel of hemodialysis therapy. Despite remarkable technological and procedural advances, VA complications continue to exert a substantial clinical, economic, and human burden, affecting morbidity, mortality, healthcare costs, and patient quality of life.

Access-related infections and fatal infections in prevalent adult patients represent one of the most serious complications of chronic hemodialysis. Evidence from large observational cohorts and meta-analyses, summarized in Table 2(A,B) [12,59,60] and encompassing more than one million patients or vascular accesses, consistently demonstrates distinct risk profiles across access types. Native arteriovenous fistulas (AVFs) carry the lowest infection rates (≈0.2/1000 fistula-days), arteriovenous grafts (AVGs) show intermediate risk (≈0.10–1.2/1000 graft-days), while central venous catheters (CVCs) are associated with the highest infection burden, with catheter-related bloodstream infection rates ranging from 0.1 to 5.15/1000 catheter-days, corresponding to a 6- to 50-fold higher risk compared with AVFs depending on catheter type and insertion site (femoral > jugular > subclavian) [61]. Importantly, CVC use is associated with more than a two-fold increase in fatal infection risk compared with AVFs, with infection rates approaching 50% at 6 months versus 3–5% for fistulas in some cohorts [59,62].

Beyond infection, complication patterns differ by access type. AVGs exhibit the highest thrombosis risk, more than four-fold higher than AVFs, largely driven by stenosis, whereas thrombosis rates for AVFs and CVCs are more comparable (~10% at 6 months). Two-year primary patency is highest for AVFs (~55%), followed by CVCs (~50%) and AVGs (~40%). Importantly, contemporary registry data indicate that both access-related infection rates infection-related mortality have declined substantially since approximately 2013, reflecting the impact of improved hygienic protocols, standardized catheter-care bundles, and quality-driven incentive programs. Although these data derive predominantly from observational studies of prevalent patients and are subject to selection bias, the consistency of findings across regions and datasets strongly supports AVFs as the preferred access whenever feasible, with individualized decisions required when fistulas are not an option.

Despite strict aseptic protocols and the use of antiseptic or citrate locking solutions, infection and dysfunction rates remain unacceptably high in tunneled catheters. Persistent challenges include catheter handling, biofilm formation, and variability in protocol adherence. Current locking strategies offer only partial protection and are limited by cost, toxicity concerns, and inconsistent efficacy [41,42,43], underscoring the need for innovative engineering solutions, such as closed connection systems, antimicrobial surfaces, and needle-free interfaces. Although antibiotic lock therapy has been shown in meta-analyses to reduce catheter-related bloodstream infections compared with heparin, its routine prophylactic use is not recommended because of the risk of promoting antimicrobial resistance, particularly with glycopeptides such as vancomycin, and it should therefore be reserved primarily for the treatment of established or proven endoluminal proven infections rather than for long-term prevention [63].

The consequences of vascular access complication extend beyond clinical outcomes. CVC-related infections and recurrent access interventions account for a disproportionate share of dialysis-related expenditures driven by hospitalizations, prolonged antibiotic therapy, and access revision [64,65,66,67].

From the patient perspective, repeated needling, pain, fear of infection or bleeding, functional limitation, and esthetic concerns make vascular access one of the most distressing aspects of dialysis therapy [46,68,69]. Addressing this multifaceted burden requires patient-centered innovations that simultaneously improve safety, durability, workflow efficiency, and quality of life.

## 3. Previous Attempts at Port Devices: Lessons from Past Failure

The concept of developing a needle-free or port-based vascular access system for hemodialysis is not new. Over the past three decades, several devices have been developed and evaluated with the aim of improving patient comfort, safety, and usability while reducing infection risk and needling-related complications. Despite promising engineering concepts, most of these systems failed to achieve widespread clinical adoption. Understanding their limitations provides valuable insights for designing the next-generation of vascular access solutions.

### 3.1. Historical Overview of Needle-Free and Port-Based Systems

Early innovations such as the Dialock^®^ and LifeSite^®^ systems (venous catheter ports) and the Hemasite^®^ arteriovenous port were developed in the late 1990s and early 2000s [15,17,19,20,70,71,72,73,74,75,76,77,78]. These devices were designed either to facilitate external catheter connections through a subcutaneously implanted port accessible percutaneously, or to replace the repeated needling required for arteriovenous access. The objectives were clear: to minimize infection risk, eliminate needling pain, improve esthetics, and simplify connection during dialysis sessions. Initial pilot studies confirmed their technical feasibility, potential clinical value, and good patient acceptance. However, available long-term outcomes were limited and generally less favorable, largely due to reported clinical complications, including skin necrosis, infection, and thrombosis, as well as economic constraints that impeded sustained use and broad clinical adoption. In historical series, implantable or port-based systems (Dialock, LifeSite, and Hemasite) tended to exhibit higher infection rates than native AV fistulas, with Dialock typically reported at approximately 1–3 infections per 1000 device-days and LifeSite at ≥2.5 per 1000 days, compared with ~0.2–0.4 per 1000 days for AVFs. Hemasite reports further described substantial thrombosis rates and a non-negligible infection burden over one-year follow-up, although comparisons are limited by heterogeneity of study design and patient selection. Overall, failure mechanisms differed by device type: port-based systems were primarily limited by skin and bloodstream infections related to transcutaneous access, whereas graft-based systems failed through a combination of local infection and venous graft stenosis. In all cases, high device and maintenance costs further restricted clinical adoption. Importantly, these early systems were developed in a technological context lacking today’s biocompatible materials, antimicrobial coatings, embedded sensors, and digital connectivity. They functioned as isolated mechanical interfaces without the ability to monitor access performance, detect early complications, or integrate into modern dialysis information systems. In parallel, we describe how recent advances in materials science, sensor technology, and digital health platforms have created a fundamentally different landscape for the development of next-generation, connected vascular access systems.

#### 3.1.1. Technical and Clinical Barriers

Most port systems required surgical or minimally invasive implantation, often involving delicate vascular manipulation and the creation of dedicated subcutaneous chambers. Over time, several complications were reported, including skin necrosis over the port site, local infections, mechanical dysfunction, venous stenosis, and thrombosis. Clinician skepticism, lack of long-term outcome data, and concerns over maintenance protocols also slowed clinical acceptance. Consequently, despite encouraging patient feedback regarding comfort and esthetics, these port-based systems failed to demonstrate clear superiority over conventional well-managed tunneled catheters or arteriovenous access in large-scale clinical practice. While the theoretical advantages of these devices, such as improved patient comfort, better esthetic outcomes, and greater ease of use for caregivers, were widely acknowledged, the risk–benefit balance ultimately became unfavorable due to a combination of surgical complexity, infection risk, high cost, and durability issues.

#### 3.1.2. Key Lessons for Future Development

Past experience underlines the need for simple, reliable, and safe vascular access connector designs that minimize infection risk, avoid complex surgical procedures, and allow easy connection under routine clinical conditions. The next generation of needle-free, closed, and digitally connected access systems should integrate lessons learned from earlier attempts, particularly regarding material selection, flow dynamics, and infection control, while leveraging modern advances in engineering, biomaterials, 3D design, and digital monitoring to ensure long-term safety, patient comfort, and sustainability.

## 4. Reimagining Vascular Access in Hemodialysis and Extracorporeal Based Therapy: The Case for Needle-Free Port Systems

Current vascular access solutions, arteriovenous fistulae/grafts or central venous catheters, remain dependent on needling or repeated mechanical handling, exposing patients to pain, tissue trauma, contamination, and human factor variability. As dialysis care progressively shifts toward more personalized, home-based, and self-care models, these limitations become more pronounced and highlight the need to reassess conventional access paradigms. Needle-free vascular access ports have emerged as a potential alternative approach, aiming to provide a closed and standardized interface between the patient and the extracorporeal blood circuit. While conceptually attractive, these devices represent a complex technological and clinical challenge, requiring careful evaluation of feasibility, safety, durability, and real-world performance before broad clinical adoption.

### 4.1. Conceptual Framework and Key Design Fundamentals

The conceptual model for next-generation vascular access port devices is grounded on several interdependent design principles. First, the system must provide a fully closed, needleless interface to avoid repeated skin puncture and reduce potential contamination pathways. Second, a secure and reproducible connection mechanism, such as a twist-lock or snap-fit docking system, is required to ensure stable handling and leak-free circuit continuity during a dialysis session. Third, the choice of biomaterials is critical and must prioritize long-term biocompatibility, anti-thrombogenic capacities, and resistance to microbial colonization. Fourth, these features may be reinforced by antiseptic locking or a shielded interface strategy intended to limit microbial ingress and biofilm formation during interdialytic periods. Finally, internal device geometry must be engineered to support adequate blood flow while minimizing shear stress, turbulence, and zones of stagnation, which are known contributors to thrombosis risk and access dysfunction. Importantly, each of these design requirements introduces technical trade-offs that may affect device complexity, reliability, and cost, underscoring the need for rigorous preclinical and clinical validation.

### 4.2. Current Technology Pathways and Prototype Configurations: The Case for Saeptum Technology

Two complementary avenues of development are emerging related to the two main access strategies: venovenous or arteriovenous access.

**Figure 1:**
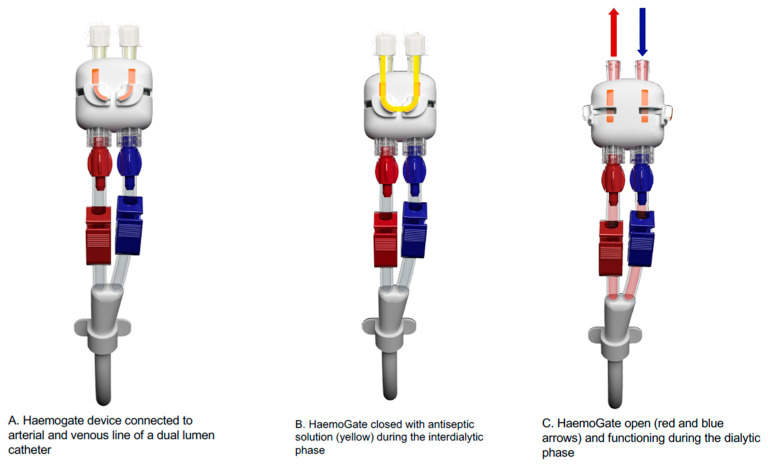
HaemoGate device as safeguard of central venous catheter connection. (**A**) HaemoGate device connected to arterial and venous line of a dual-lumen catheter; (**B**) HaemoGate closed with antiseptic solution (yellow) during the interdialytic phase; (**C**) HaemoGate open (red and blue arrows) and functioning during the dialytic phase.

The **first category refers to venovenous access prototypes** (e.g., HaemoGate): dual-lumen protected access ports serving as an evolution of temporary untunneled and permanent tunneled catheters, consisting of an external add-on connection port attached to the existing catheter hubs via a luer-lock system, offering fully protected access to both catheter lines. By eliminating direct hub manipulation during dialysis handling, they reduce the risk of contamination, and the integrated antiseptic locking reservoir further decreases the risk of infection during the interdialytic period. An illustration of this HaemoGate device is shown in Figure 1, connected to the arterial and venous lines of a dual-lumen catheter, showing the closed (locked with antiseptic) and open configurations during an HD session. A feasibility and safety clinical study is currently ongoing in patients with tunneled CVCs.

**Figure 2:**
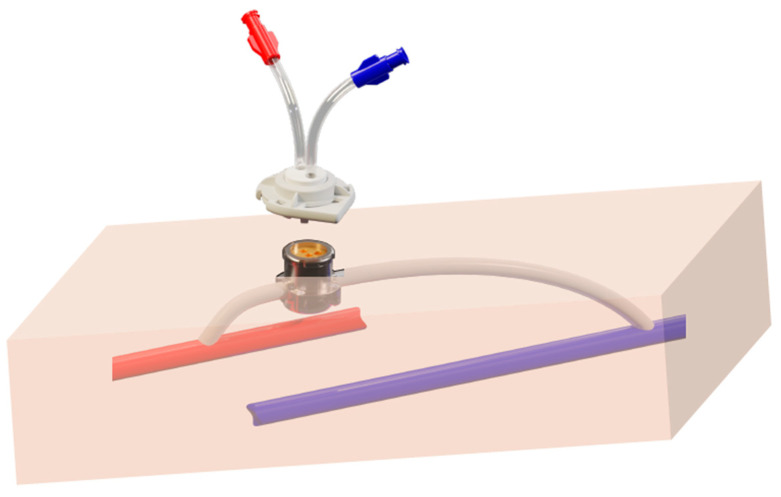
Subcutaneous arteriovenous port device (SHIVAT) implanted between the artery and the vein (PTFE graft, with the access port emerging through the skin). The disposable twist-lock connector, equipped with arterial and venous tubing segments, docks onto the access port and allows secure attachment to standard hemodialysis bloodlines.

The **second category refers to arteriovenous needle-free ports** (e.g., Safe Hemodialysis Implantable Vascular Access Technology (SHIVAT) concept), which rely on a subcutaneous surgically implanted port integrated into a graft or vascular conduit. The system emerges at the skin surface to enable percutaneous docking without needles, by means of a disposable twist-lock cap, while providing high blood flow suitable for hemodialysis. An illustration of this AV port system is shown in Figure 2. A pilot animal study has been conducted to validate the concept and assess its surgical feasibility and functional performance [79]. These developments are progressing through bench testing, followed by bioengineering validation and early in vivo studies in animal and human settings. Although preliminary results suggest favorable flow dynamics, sealing integrity, and handling ergonomics, these findings require confirmation in larger and longer-term clinical evaluations, as the technology remains experimental.

### 4.3. Potential Advantages, Risks, and Limitations Compared with Conventional Systems

In theory, port-based vascular access systems could improve safety and usability by standardizing the connection process and reducing reliance on repeated needling or manual catheter handling. By eliminating repeated skin punctures, they may enhance patient comfort and reduce cumulative tissue trauma, particularly in patients with fragile skin, extensive scarring, or needle-related phobias. Standardizing the connection technique also reduces inter-operator variability and facilitates self-care or home dialysis programs, where simplicity and reproducibility are essential. However, these potential advantages must be weighed against several important limitations. Implantable port systems introduce surgical complexity and may carry specific risks related to implantation, explantation, infection, thrombosis, or device failure. Long-term performance under repeated high-flow extracorporeal use remains insufficiently documented. The risk of stenosis and thrombosis affecting the vascular graft used or conduct supporting arteriovenous devices (e.g., SHIVAT) require evaluation in adequately powered, long-term studies. In addition, the potential for deep-seated infection or biofilm formation within implanted components may present challenges distinct from those seen with conventional accesses. Economic considerations, including device cost, training requirements, and reimbursement pathways, may further limit widespread adoption.

**Figure 3:**
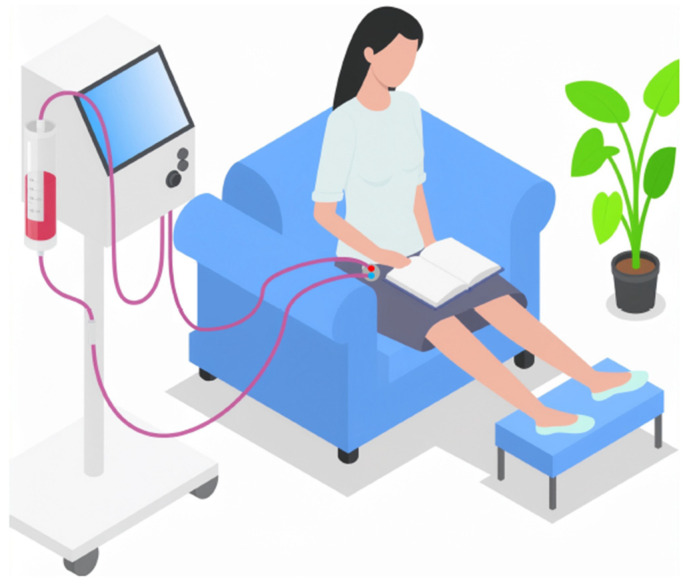
Example of a home hemodialysis patient connected to a SHIVAT port located in the femoral area, providing greater freedom by freeing the use of the arms.

**Figure 4:**
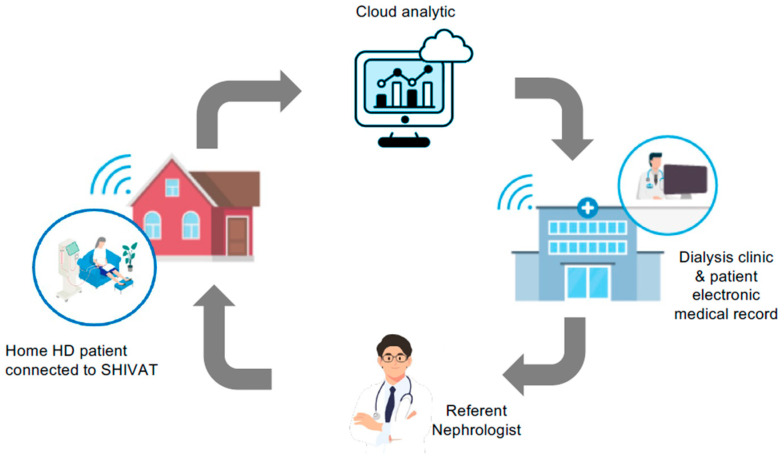
Remote monitoring of the SHIVAT port device for home HD patients, with online performance data analyzed and transmitted to the caregiver and nephrologist.

**Figure 5:**
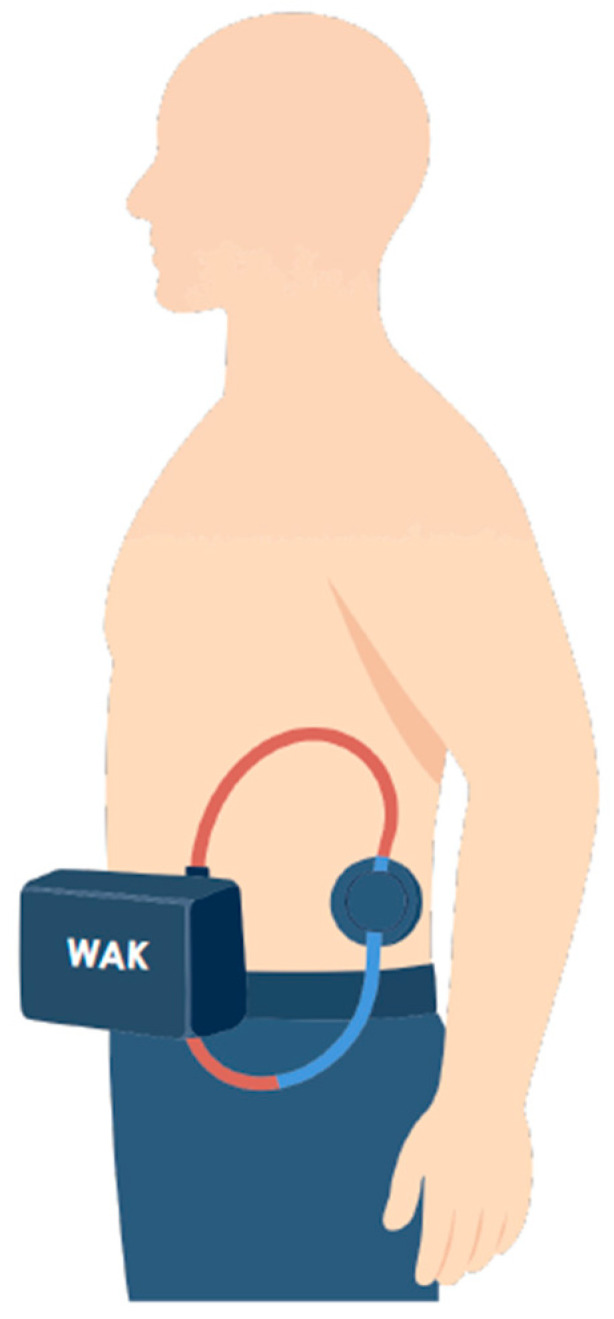
Wearable artificial kidney connected to the SHIVAT port positioned on the abdominal wall.

The ability to implant ports at alternative anatomical sites, such as the iliac or femoral vascular networks, could expand access options in selected patients and facilitate arm-free dialysis configurations (Figure 3). Nonetheless, these approaches require careful patient selection and raise additional concerns related to infection risk, mobility, and long-term vascular preservation. In this context, SHIVAT-based technology also offers the opportunity for continuous or remote monitoring of vascular access function and integrity, enabling early detection of dysfunction, as schematically illustrated in Figure 4. Looking ahead, vascular access port technology may also support the development of wearable or hybrid extracorporeal therapies; however, access reliability and safety remain critical bottlenecks that must be addressed before such applications can be realized at scale [80,81,82,83,84]. Figure 5 illustrates a conceptual a belt-type wearable artificial kidney connected to an iliofemoral arteriovenous port.

### 4.4. Roadmap Toward Clinical Translation

Successful clinical translation of a vascular access port system requires a phased strategy spanning preclinical validation, regulatory approval, economic validation, and clinical adoption. Development begins with engineering verification and bench testing to assess feasibility, flow performance, structural integrity, and fatigue resistance, followed by validation in animal studies to confirm performance and durability, biocompatibility, and infection control. Early feasibility has been demonstrated in animal models and first-in-human studies of external dual-lumen catheter-based prototypes.

Regulatory pathways will follow requirements for high-risk implantable devices (EU-MDR Class III/FDA PMA), with emphasis on long-term performance, structural integrity, microbial ingress prevention, and risk mitigation of device-specific drawbacks. Mid- and long-term clinical trials will be necessary to support regulatory submissions, compare performances with existing access options, and substantiate clinical benefit. In parallel, demonstration of health economic value will be essential in real-world settings, particularly reductions in infection-related hospitalizations, nursing workload, consumable use, access revisions, and missed dialysis sessions.

Widespread adoption will ultimately rely on human factors engineering, standardized training, and structured implementation pathways. A stepwise rollout from in-center dialysis units to assisted and self-care home settings will be essential to ensure safe use, reproducibility, and durable integration into chronic kidney replacement care.

## 5. Future Directions and Research Needs

Looking ahead, the next-generation needle-free and digitally enabled vascular access systems should be evaluated using a stepwise and evidence-based framework. Several enabling technologies, such as miniaturized pressure and flow sensors, wireless data transmission, and antimicrobial or bioactive materials, are already available and are beginning to be incorporated into implantable medical devices. Their integration into vascular access ports offers the potential to provide continuous or intermittent monitoring of access function and integrity. In contrast, more advanced concepts, such as embedded nano-biosensors for early detection of subclinical infection, leakage, or biofilm formation, as well as automated decision support systems, remain largely at the research and prototyping stage and require substantial preclinical and clinical validation before clinical use can be considered. These evolving technologies may be particularly relevant for emerging portable, wearable, or hybrid extracorporeal dialysis systems currently under development or early clinical evaluation [80,82,85] for which access reliability and low-burden handling are essential. However, their feasibility, safety, and added value must be demonstrated rather than assumed.

Beyond technology performance alone, future evaluations must incorporate patient-reported outcome measures (PROMs) to capture comfort, usability, confidence, anxiety reduction, and patient’s quality-of-life benefits, domains that are traditionally rarely captured with access assessments. Finally, rigorous research frameworks will be needed, combining well-designed randomized controlled trials with pragmatic real-world implementation studies to assess safety, infection prevention, device longevity, and economic impact. Together, these steps will help define the clinical, human, and economic value proposition of intelligent, needle-free vascular access solutions and accelerate their translation into routine dialysis practice.

## 6. Conclusions

Future advances in hemodialysis will depend not only on improving dialysis efficiency, but also on reducing the clinical, organizational, and patient burden associated with vascular access. Needle-free, digitally connected port-based access systems represent a potentially valuable alternative to conventional fistulas, grafts, and catheters by addressing key limitation related to repeated needling, open handling and variability in aseptic practice.

However, the clinical role of these technologies remains to be defined. Important uncertainties persist regarding long-term durability, risks of thrombosis and deep-seated infection, surgical complexity, cost-effectiveness, and integration into existing care pathways. Addressing these limitations will require stepwise clinical development, including comparative effectiveness studies against current access standards, long-term surveillance of safety and performance, and rigorous health economic evaluations.

Future research should focus on identifying appropriate patient populations, optimizing device design and implantation strategies, and assessing usability in both in-center and home dialysis settings. Only through such evidence-based evaluation can port-based vascular access systems be appropriately positioned within the therapeutic life plan of patients and potentially contribute to the evolution of extracorporeal and wearable renal replacement therapy [81]. At present, however, these technologies should be considered experimental and complementary rather than replacements for established vascular access modalities.

## Figures and Tables

**Table 1:** Comparative summary of vascular access modalities with respect to patient experience, workload, suitability for home-based care, and specific risks.

Access Type	Patient Comfort	Needling Stress	Infection Risk	Setup Time	Staff Required	Consumables	Home HD Suitability	Self-Care Suitability	Notes Risks
AV Fistula	High	High	Low	5–10 min	1	Low	High	High	Reference
AV Graft	Moderate	High	Moderate	5–10 min	1	Low	Moderate	Moderate	Higher Failure Risk
Tunneled CVC	Low	Absent	High	15–20 min	2	High	Low	Low	Higher infection Rate
Needle-free Port (future)	High	Absent	Very Low	<5 min	1	Low	Very High	Very High	Vascular Access 4.0

**Table 2:** (**A**,**B**) Systematic review of recent major studies comparing complication profiles of hemodialysis vascular access types (AVF, AVG, and CVC), including infection rates, thrombosis risk, primary patency, and mortality risk.

A							
Article Reference	Study Type		Total N (Participants & Accesses)	Arms (AVF/AVG/CVC)		Follow-Up Timepoint	Exposure Metric
Pastan 2002 KI [12]	Retrospective cohort (prevalent HD patients)		7403	AVF, AVG, cuffed catheter, noncuffed catheter (catheters analyzed together for mortality models)		10–11 months	Variable follow-up; logistic model; no patient-time
Colville 2006 ICHE [60]	Retrospective surveillance (single-center)		≈50	AVF (51%), AVG (10%), permanent tunneled cuffed CVC (37%), temporary non-tunneled CVC(≈2%)		12 months	14,528 patient-days (true exposure)
Ravani 2013 JASN [62]	Systematic review + meta-analysis		586,337	Comparisons across AVF, AVG, CVC (tunneled/any)		Varied across included cohorts	Meta-analysis; heterogeneous follow-up; no patient-time
Almasri 2016 JVS [59]	Systematic review + meta-analysis		875,269	AVF, AVG, tunneled cuffed catheter (and others)		Key pooled outcomes at ~2 years	Fixed timepoints (1–2 years); no patient-days
**B**							
**Article** **Reference**	**Outcome domain**	**Metric timepoint**		**AVF**	**AVG**	**CVC**	**Effect vs. AVF**
Pastan 2002 KI [12]	Mortality	Adjusted OR for all-cause death		Reference	**OR 1.1** vs. AVF (0.9–1.4)	**OR 1.4** (1.1–1.9) vs. AVF; **OR 1.3** (1.1–1.6) vs. AVG	CVC vs. AVF OR **1.4**
Pastan 2002 KI [12]	Mortality (infection-related)	Adjusted OR for infection-related death		Reference	**OR 1.4** vs. AVF (0.6–2.8)	**OR 3.0** (1.4–6.6) vs. AVF; **OR 2.2** (1.4–3.6) vs. AVG	CVC vs. AVF OR **3.0**
Colville 2006 ICHE [60]	Infection	Confirmed BSI incidence (per 1000 patient-days)		**0.4**	**2.86**	Permanent **4.02**; Temporary **20.2**	RR vs. AVF: AVG **7.15**; perm CVC **10.05**; temp CVC **50.50**
Ravani 2013 JASN [62]	Mortality	All-cause mortality (pooled RR)		Reference	**RR 1.18** (1.09–1.27) vs. AVF	**RR 1.53** (1.41–1.67) vs. AVF; **RR 1.38** (1.25–1.52) vs. AVG	AVG vs. AVF RR **1.18**; CVC vs. AVF RR **1.53**
Ravani 2013 JASN [62]	Infection	Fatal infection (pooled RR)		Reference	**RR 1.36** vs. AVF (1.17–1.58)	**RR 2.12** (1.79–2.52) vs. AVF; **RR 1.49** (1.15–1.93) vs. AVG	AVG vs. AVF RR **1.36**; CVC vs. AVF RR **2.12**
Almasri 2016 JVS [59]	Patency	Primary patency (pooled) at 2 years		**0.55** (0.52–0.58)	**0.40** (0.35–0.44)	**0.50** (0.41–0.61) at 86 weeks	Crude ratio: AVG/AVF = **0.73**; CVC/AVF ≈ **0.91**
Almasri 2016 JVS [59]	Patency	Secondary patency (pooled)		**0.63** (0.59–0.67) at 2 years	**0.60** (0.55–0.65) at 2 years	**0.43** (0.27–0.69) at 39 weeks (single study)	Crude ratio: AVG/AVF ≈ **0.95**
Almasri 2016 JVS [59]	Infection	Access infection rate (pooled)		**0.02** (0.01–0.04)	**0.13** (0.10–0.17)	**0.16** (0.08–0.34) at 59 weeks	Crude ratio: AVG/AVF = **6.5**; CVC/AVF = **8.0**
Almasri 2016 JVS [59]	Mortality	Mortality at 2 years (pooled incidence)		**15%**	**17%**	26%	Crude ratio: AVG/AVF = **1.13**; CVC/AVF = **1.73**
Almasri 2016 JVS [59]	Mortality	Comparative effect (adjusted)		Reference	**HR 1.278** vs. AVF (1.139–1.435)	Reported higher than AVF/AVG	AVG vs. AVF HR **1.278**

## Data Availability

No new data were created or analyzed in this study.
